# Supplementary material for: Identification and functional analysis of non-coding regulatory small RNA FenSr3 in Bacillus amyloliquefaciens LPB-18
Source: PeerJ. 2023 May 15;11:e15236. doi: 10.7717/peerj.15236 (PMC10194069; doi:10.7717/peerj.15236)
Supplement: Supplemental Information 4 [file peerj-11-15236-s004.zip › KO/CK-vs-T1_map/map04151.html]

KEGG PATHWAY: PI3K-Akt signaling pathway - Reference pathway


|  |  |
| --- | --- |
| **PI3K-Akt signaling pathway - Reference pathway** |  |

[
Pathway menu
| Organism menu
| Pathway entry
| Show description
| User data mapping
]

The phosphatidylinositol 3' -kinase(PI3K)-Akt signaling pathway is activated by many types of cellular stimuli or toxic insults and regulates fundamental cellular functions such as transcription, translation, proliferation, growth, and survival. The binding of growth factors to their receptor tyrosine kinase (RTK) or G protein-coupled receptors (GPCR) stimulates class Ia and Ib PI3K isoforms, respectively. PI3K catalyzes the production of phosphatidylinositol-3,4,5-triphosphate (PIP3) at the cell membrane. PIP3 in turn serves as a second messenger that helps to activate Akt. Once active, Akt can control key cellular processes by phosphorylating substrates involved in apoptosis, protein synthesis, metabolism, and cell cycle.

|  |  |  |
| --- | --- | --- |
| Reference pathway | 184% 150% 122% 100% 82% 67% 55% | 图片下载 |
